# Practitioners’ experience of the working alliance in a blended cognitive–behavioural therapy intervention for depression: qualitative study of barriers and facilitators

**DOI:** 10.1192/bjo.2022.546

**Published:** 2022-07-25

**Authors:** Asmae Doukani, Caroline Free, Ricardo Araya, Daniel Michelson, Arlinda Cerga-Pashoja, Ritsuko Kakuma

**Affiliations:** Faculty of Epidemiology and Population Health, London School of Hygiene and Tropical Medicine, London, UK; Health Service and Population Research Department, King's College London, UK; School of Psychology, University of Sussex, Brighton, UK

**Keywords:** Working alliance, mental health practitioner, blended cognitive–behavioural therapy, qualitative research, e-mental health

## Abstract

**Background:**

Digital technologies have been widely acknowledged as a potentially useful resource for increasing mental healthcare access. The working alliance is a key influence on outcomes in conventional psychotherapy, but little is known about therapists’ experiences of forming an effective working alliance in blended interventions that involve in-person psychotherapy and a digital programme.

**Aims:**

To investigate psychological well-being practitioners’ (PWPs’) experiences of the working alliance in a trial of blended cognitive–behavioural therapy (b-CBT) for depression. Trial registration ISRCTN12388725.

**Method:**

Semi-structured qualitative interviews were conducted with 13 PWPs who delivered b-CBT in a two-arm, non-inferiority randomised controlled trial investigating the effectiveness of b-CBT compared with face-to-face CBT. Thematic analysis was used to analyse the data.

**Results:**

Participants reported four facilitating factors when building and maintaining a working alliance in b-CBT: having more time to deliver treatment, access to a wider toolkit, capacity to tailor components of b-CBT and receiving appropriate training and support. Participants also identified four barriers to building and maintaining a working alliance: time and resource constraints, usability challenges, limited flexibility to tailor the digital programme to patients’ needs and lack of confidence in delivering b-CBT.

**Conclusions:**

Our study is the first specifically to investigate practitioners’ perceived facilitators and barriers to forming a working alliance in b-CBT for depression. Findings suggest that PWPs’ experiences of the working alliance can be improved by: accounting for the time required to deliver b-CBT in service workflows to reduce time pressures; increasing opportunities to tailor the digital programme through offering transdiagnostic tools and adaptable features; and providing appropriate b-CBT training and technical support.

Digital technologies have been widely acknowledged as a potentially useful resource for increasing access to mental healthcare, offering the promise of affordable, evidence-based interventions at scale, as well as opportunities to augment and extend treatments in new and innovative ways.^1–6^ Digital interventions such as internet-based cognitive–behavioural therapy (i-CBT) have become increasingly popular in the past decade. i-CBT can be implemented with different levels of human support, ranging from no/minimal support, referred to as unguided i-CBT, to regular in-person sessions, referred to as blended CBT (b-CBT). Although there is some evidence to suggest that b-CBT is effective in treating depression compared with no treatment,^7^ very little is understood about therapists’ experiences of developing and maintaining a central concept of engagement in psychotherapy, called the working alliance.^8,9^

## Working alliance

Edward Bordin^10,11^ conceptualised a tripartite theory of the working alliance, consisting of three common factors that apply to most, if not all psychotherapeutic approaches: goals, the task and the bond. Goals or goal setting involves the collaborative effort between the therapist and patient to identify what the patient wants to achieve through therapy. Goals are generally established at the start of therapy and subsequently frame the activities (i.e. the task) selected in treatment. Goals are also reviewed and fine-tuned throughout therapy to ensure that they remain relevant to the patient. The task refers to an agreed therapist–patient exchange and activities that specify how the patient's goals can be achieved. The patient–therapist collaboration involves the active involvement of the patient in the selection of the task to ensure that it is relevant to their goals, with the therapist considered a major source of task selection owing to their clinical expertise and insights. Finally, the bond refers to a partnership that stems from shared activities and compatibility between therapist and patient. Bordin^10,11^ describes shared activity as a sense of ‘common commitment and shared understanding of the activities carried out in therapy’, while compatibility is described in terms of liking, trusting and respecting one another.

The working alliance is important because it has been found to predict treatment outcomes for a range of psychological interventions, including conventional face-to-face CBT for depression.^12–14^ Although some efforts have been directed towards understanding the working alliance from the patient's perspective both quantitively^15–17^ and qualitatively,^18^ to our knowledge none have specifically explored therapists’ perspectives of the working alliance in i-CBT or b-CBT. In a previous study^18^ we examined the patient's experience of the working alliance and found that, while Bordin's^10,11^ bond, goals and task largely remained relevant in a b-CBT setting, a fourth dimension called ‘usability heuristics’ underscored the impact of the digital programme on the working alliance. Usability heuristics was defined as the use of digital technologies to promote active engagement with the digital programme, through higher levels of accessibility, immediacy, ease of use, aesthetic appeal and opportunities for self-directed treatment. These findings indicate that the patient's working alliance demands are directed not only towards the therapist, who according to the research is largely responsible for maintaining the bond, goals and task, but also towards the digital programme.

A study by Titzler and colleagues^19^ that explored therapists’ general experiences of implementing b-CBT reported that a lack of autonomy in how patients used the intervention, a ‘one size fits all’ approach and persistent technical problems hindered the patient–therapist alliance.^19^ These findings indicate that programme-related factors may influence how the working alliance is perceived.

## Rationale and aim

There appears to be some evidence to suggest that patients' experience of the working alliance may be different in b-CBT, and that programme-related aspects of implementing and delivering b-CBT (e.g. a lack of autonomy and poor usability) can have a negative impact on therapists’ perceptions of the patient–therapist alliance.^18,19^ These findings warrant an in-depth examination of therapists’ perceptions of managing the working alliance in a b-CBT context. Such insights can be used to optimise the working alliance and the implementation of b-CBT. Based on the reasoning oulined, our study aims to qualitatively examine therapists’ experience of the working alliance in a b-CBT intervention for depression, on the E-COMPARED trial.

## Method

### Design of trial

This study was nested in the E-COMPARED project, a two-arm, non-inferiority randomised controlled trial investigating the effectiveness of b-CBT compared with treatment as usual (TAU) across nine European countries.^22^ The study was conducted in the UK site and it enrolled patients aged 18 years or older who met the DSM-IV diagnostic criteria for major depressive disorder.

In summary, inclusion and exclusion criteria^22^ applied on the E-COMPARED trial were as follows.^22^

Inclusion criteria: aged 18 years or older; meeting diagnostic criteria for major depressive disorder; score of 5 or higher on the Patient Health Questionnaire-9 (PHQ-9).

Exclusion criteria: high risk of suicide; psychiatric comorbidities (bipolar affective disorder, obsessive–compulsive disorder, psychotic illness and substance dependence); currently receiving psychological treatment; unable to speak or write English; no access to fast internet connection; does not have an android smartphone or is not willing to carry one provided by the research team.

Further information on the E-COMPARED trial can be found in the trial protocol.^22^

### Participants

Eligible participants were low-intensity psychological well-being practitioners (PWPs) recruited from six Improving Access to Psychological Therapies (IAPT) services across the UK. IAPT services aim to improve access to, and delivery of, evidence-based psychological interventions within the National Health Service. IAPT services provide evidence-based treatments for adults with a range of anxiety and depressive disorders, and with comorbid presentations. The PWP workforce typically provide low-intensity, short-term, evidence-based treatments using cognitive–behavioural principles and in accordance with the National Institute for Health and Care Excellence (NICE) guidelines.^20^ A ‘low-intensity’ PWP generally uses self-help material and engages in 6 h or less of contact with patients, with each session being around 30 min or less.^21^ PWPs who delivered at least one face-to-face session on the b-CBT arm of the E-COMPARED trial were emailed a study information sheet before they were followed up and booked in for an individual interview or a focus group discussion (FGD) at the service in which they worked. The study aimed to maximise diversity in the sample, based on gender, age, years of experience, service location and number of participants seen in the b-CBT arm. Data collection took place between July 2016 and June 2017.

### Ethics approval and informed consent

The authors assert that all procedures contributing to this work comply with the ethical standards of the relevant national and institutional committees on human experimentation and with the Helsinki Declaration of 1975, as revised in 2008. All procedures involving human participants/patients were approved by the Health Research Authority's Ethics Committee on 17 April 2015 (REC reference: 15/LO/0511) and the London School of Hygiene and Tropical Medicine Research Ethics Committee on 9 June 2015 (Ethics Ref: 9409). Therapists provided written informed consent prior to participation in the individual interviews and the focus group interviews.

### The b-CBT intervention

Treatment conditions on the E-COMPARED trial consisted of b-CBT and TAU for depression. The b-CBT was delivered by PWPs in the clinic and supported by i-CBT modules that were ideally completed outside of the clinic. The i-CBT Moodbuster^22^ intervention includes four mandatory modules (psychological education, behavioural activation, cognitive restructuring and relapse prevention) and two optional modules (physical exercise and problem-solving). Moodbuster is supplemented with a mobile app, which allows patients to rate and visualise their mood and receive reminders of their scheduled behavioural activation activities. It was also used by the research team to collect ecological momentary data at the start and end of treatment. Trial participants in the b-CBT group were offered a target number of 11 alternate sessions, six face-to-face in the clinic and five via Moodbuster and the mobile app (hereafter, Moodbuster and the app are referred to collectively as the digital programme). PWPs were required to access a therapist portal on the Moodbuster platform to monitor patient progress on the modules, and mood and depression symptom ratings. PWPs were also able to book appointments and send direct messages to the patients between clinic sessions via a therapist portal.

### Data collection tools

Qualitative data were collected using a mixture of individual interviews and FGDs, which were conducted according to semi-structured topic guides.^23^ Areas of inquiry were loosely guided by Bordin's^10,11^ theory of the working alliance. The topic guides for both individual interviews and FGDs aimed to understand PWPs’ experiences of building a working alliance, covering a broad range of questions pertaining to the implementation/delivery of b-CBT and the working alliance. Individual interviews (topic guide 1) aimed to gain in-depth insights of the working alliance in b-CBT and FGDs (topic guide 2) explored shared experiences of forming a working alliance in b-CBT^23^ (the topic guides are shown in the supplementary material available at https://doi.org/10.1192/bjo.2022.546). The FGDs also enabled efficient data collection during a time-sensitive period of the trial. For pragmatic reasons two PWPs (P03 and P04) were involved in both the individual qualitative interviews and the FGDs. Steps were taken to ensure that the weighting of their perspectives did not skew the data, by identifying them as a single source during the data analysis and reporting of the findings. All individual interviews and FGDs were audio-recorded using an Olympus digital voice recorder WS-852 and were transcribed verbatim.

### Analysis

**Fig. 1:**
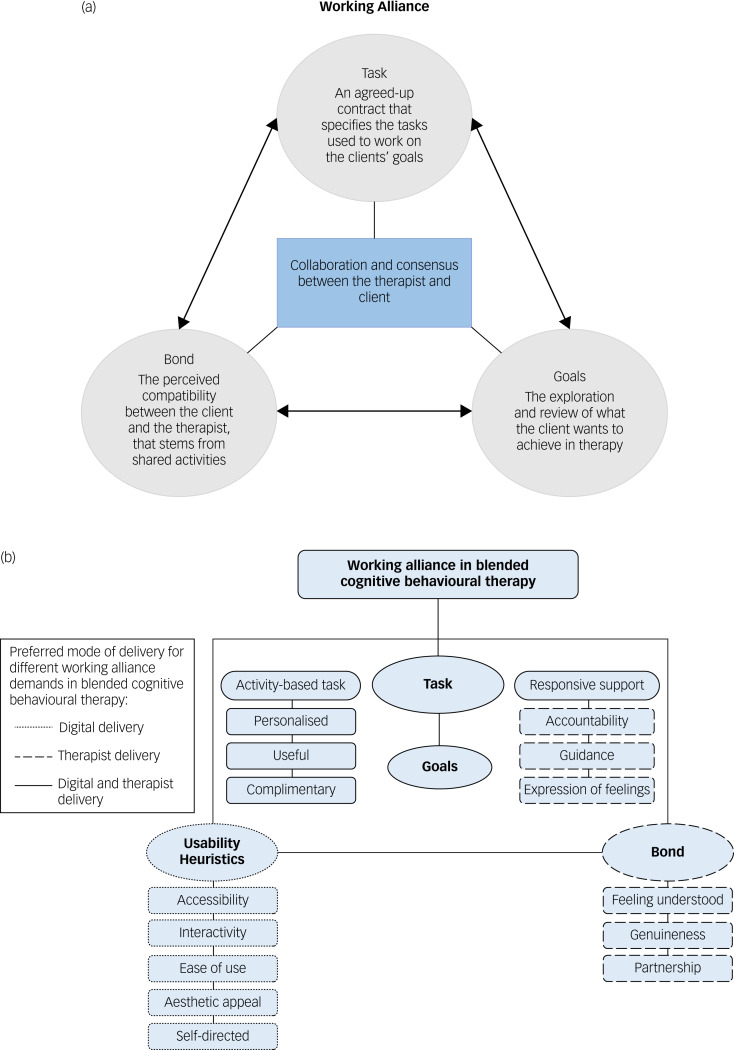
The working alliance frameworks used to guide the topic guide. (a) Diagrammatic overview of Bordin's working alliance theory.^10,11^ (b) Conceptual framework of the working alliance in a blended cognitive behavioural therapy for depression^18^ (reproduced with permission of Asmae Doukani).

Qualitative data from individual interviews and FGDs were combined and analysed using NVivo 12^24^ on a personal computer. Thematic analysis was adopted owing to its theoretical flexibility and potential for in-depth description.^25^ We took a primarily deductive approach to generate codes and themes, reflecting the study's overarching aims and drawing on Bordin's^10,11^ aforementioned theory of working alliance and a previous study by our group^18^ which used a qualitative design to formulate a conceptual framework of the working alliance in b-CBT for depression (Fig. 1). Data were also analysed inductively, by staying open to factors that positively or negatively affected the PWPs’ experience of the working alliance. Thematic analysis involved reading the transcripts to enable familiarisation with the data. Data were then coded line by line and later reviewed to identify patterns to generate themes. Based on the emerging data, each theme was then categorised as either a facilitator or barrier to forming a working alliance. Themes were then reviewed to ensure that they were relevant to the working alliance.^25^ Once a final list of themes and categories was developed, theme names were refined and each theme was described.^25^ The qualitative analysis was conducted by author A.D. in full. Different phases of data analysis were partially reviewed and/or analysed by two co-authors to check that data were accurately coded, to maintain objectivity and avoid bias. This was done by checking the codes against the data to ensure that supporting quotations accurately depicted the loose deductive frameworks outlined in Fig. 1. Having several people involved in the coding process also helped bring different perspectives and interpretations to the analysis.^26^ After A.D. coded two individual interview transcripts, C.F. reviewed all codes and supporting quotations. Following the completion of the initial coding phase by A.D., R.K. analysed a portion of the data consisting of one individual interview and one FGD. R.K.'s findings were compared with A.D.'s in a meeting to discuss similarities and discrepancies. Later stages of analysis involving the further development of themes were overseen by R.K. and in consultation with other co-authors. Themes were also discussed and refined over four meetings/consultations with co-authors, who were mental health and primary care service experts and who were very familiar with the concept of the working alliance.

## Results

### Participant characteristics

Out of the 29 PWPs approached about the study, 13 provided consent and participated in FGDs (*n* = 9), individual interviews (*n* = 2) and a combination of both (*n* = 2). Participants’ mean age was 26.6 years (s.d. = 2.55) and they had worked as PWPs for a mean of 35.1 months (s.d. = 14.19) (see Table 1 for full participant characteristics).

**Table 1:** Participant characteristics

Characteristic^a^	
Age, years (s.d.), range	26.6 (2.55), 23–31
Female gender, *n* (%)	8 (61.5)
Experience, years (s.d.), range^b^	35.1 (14.19), 12–53 months
Patients on b-CBT, *n* (s.d.), range	2.7 (1.73), 1–6 patients
Qualifications (*n* = 10)	
Bachelor's degree, *n* (%)	4 (40)
Master's degree, *n* (%)	4 (40)
Professional diploma, *n* (%)	2 (20)
Interviews, *n*^c^	
Individual (topic guide 1)	4
FGD1 (topic guide 2)	5
FGD2 (topic guide 2)	2
FGD3 (topic guide 2)	3
Individual (topic guide 2)	1
Site	
Site A	9 (69.23)
Site B	4 (30.77)

b-CBT, blended cognitive–behavioural therapy; FGD, focus group discussion.

a. Data for age, gender, years of experience and qualification were based on 10 participants, as 3 participants from FGD1 (*n* = 1) and FGD3 (*n* = 2) did not provide demographic data.

b. Years of experience in role as a psychological well-being practitioner (PWP).

c. One individual interview was conducted using topic guide 2, as the participant could not attend a focus group.

### Thematic analysis

**Fig. 2:**
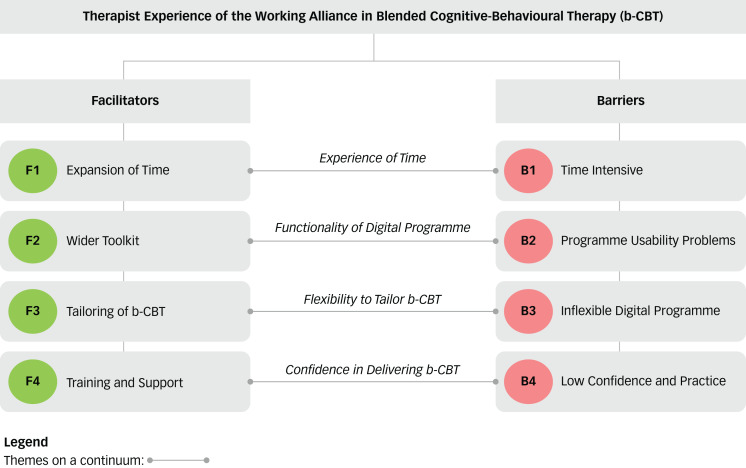
Therapist-reported facilitators and barriers in building a working alliance in a blended cognitive behavioural therapy intervention. F, facilitator; B, barrier.

Eight themes were identified and grouped as facilitators and barriers in building a working alliance. The facilitators were: (F1) expansion of time; (F2) wider toolkit; (F3) tailoring of b-CBT; and (F4) PWP training and support. An additional four themes were identified as barriers: (B1) time-intensive; (B2) usability problems; (B3) inflexible digital programme; and (B4) low confidence and practice. The analysis also identified four higher-order, cross-cutting categories that drew links between facilitators and barriers: experience of time (which encompasses F1 and B1), functionality of the digital programme (F2 and B2), flexibility to tailor b-CBT (F3 and B3) and confidence in delivering b-CBT (F4 and B4). See Fig. 2 for a diagrammatic representation of the facilitators, barriers and higher-order categories.

### Time in b-CBT

Most PWPs fed back that b-CBT provided opportunities to extend patients’ time in treatment but was time intensive, highlighting facilitators and barriers in building and maintaining all elements of the working alliance (bond, goals and task).

#### F1 Expansion of time

On one hand, PWPs reported that integrating the digital programme to in-person therapy extended the time of the patients’ treatment course (i.e. hours that patients were engaged with the treatment), thereby also increasing patients’ dosage of the task. They described the computerised modules as time-saving, removing the pressure of completing tasks during face-to-face sessions, which provided additional time for PWPs to talk to their patients and better reflect on treatment processes, in aid of further developing the patient–therapist bond.

‘I think it's quite a subtle change in [the] therapeutic relationship when you're doing blended therapy to face-to-face, I think because ultimately that space is a lot more effective […], it's not quite so structured but you have to get through x, y and z in this time, you've got a bit more time to reflect on things, and it's [it gives you] a bit more agency … ’ (P03)

#### B1 Time intensive

On the other hand, PWPs also reported that implementing b-CBT was time intensive, as additional time was required to familiarise themselves with the content of the programme and prepare for each session by reviewing the patients’ progress online. They also fed back that adapting the treatment to patients’ needs was more time-consuming in a blended format. The time required to deliver the b-CBT intervention did not fit in with the service flow, thus putting additional strain on the PWPs’ ability to learn and apply the task as intended:

‘I think it was a steep learning curve for us to try to remember and to get our heads around the programme and feel confident and competent enough with it that if a patient came to us saying “Oh, I was looking at this section or that section – I don't understand what it is. Can you explain things?” Like that we'd have to kind of know what they were talking about not quickly looking it up.’ (P04)

‘It did [have an impact on my case-load] because with the c-CBT [computerised-CBT] reviews, the online reviews, they weren't counted as part of our target [….] So I suppose the online review was very much a case of trying sometimes to squeeze it in around the rest of your workload.’ (P015)

‘ … if you think of the IAPT framework, our service like we get about 1200 referrals a month […], face-to-face we're seeing people for about 6 to 8 weeks and then they're being moved on or stepped up, whereas this would then delay it, you know, to 12 weeks. So it's just going to create a massive bottleneck where people are going to have to wait a lot longer to be seen, which could cause some problems there just because we're dealing with such a huge volume of referrals.’ (P012)

### Functionality of the digital programme

Some PWPs experienced the functionality of the digital programme as positive, enhancing their therapeutic toolkit in aid of addressing the task and goals, whereas others reported poor functionality that limited the patients’ engagement with the digital programme, which is essential for accessing the task (including tasks that are key to the goal-setting process) agreed on between the PWP and the patient.

#### F2 Wider toolkit

PWPs reported that the digital programme provided them with a wider toolkit to build a working alliance (e.g. ability to message the patient between sessions, track the patient's mood throughout treatment) and help the patient engage with the goals and task. Features of i-CBT provided new mechanisms of supportive accountability, allowing PWPs to monitor adherence to the task via a therapist portal. Other PWPs said that they were able to extend their presence outside of the clinic by using the messaging feature of the programme between sessions. PWPs also reported that the addition of i-CBT enabled a more systematic and comprehensive coverage of the task than would have otherwise been possible, given that patients were required to complete all core modules on the programme. PWPs also stated that the blended format provided the patients with additional opportunities to consolidate what was learned through the agreed task and goals, across different modes of delivery:

‘I think it gives patients something to sort of focus on in between the sessions. I think it kind of reinforces those techniques so I really like the reminders on the phone, sort of looking at kind of diaries and things in more of an interactive way … ’ (P015)

‘I guess I would say […] I found having the session once a fortnight good for the patients, it was great for them, I think they had a bit of extra time to consolidate learning so you could really see that improvement over 2 weeks. Whereas our standard sessions are weekly so sometimes they haven't had quite as much time to practise the techniques that were covered in session.’ (P012)

#### B2 Usability problems

Although most participants said that the programme was generally user-friendly, concerns were expressed in relation to aspects of the digital programme's functionality. Some PWPs noted that technical issues or poor programme usability, particularly those that could not be readily fixed (e.g. malfunctioning mood-rating alerts on the mobile app that sounded multiple times a day and could not be turned off), may have had a negative impact on the working alliance. However, more straightforward technical issues (e.g. patients forgetting their ID) caused less concern for PWPs. Persistent usability problems therefore appeared to threaten ‘usability heuristics’ – that is, the patients’ ability to use the digital programme to enable engagement, self-discovery and autonomous problem-solving in blended b-CBT – thereby creating barriers to addressing the working alliance needs in relation to accessibility, ease of use and self-directed use. Persistent usability problems that affect the patient's access to the tasks also hindered the PWPs’ ability to deliver the task and goals as intended or to maintain a bond in respect to keeping the patient motivated and engaged in treatment:

‘One person had practical problems with the phone in that it wasn't going off as often as it should, and then he wasn't sure when he should and shouldn't be doing it, and I did flag it with [researcher's name], but it's still, it's like confusing and he kept asking me these questions about it, and like having to forward it on to [researcher's name], and it was like I got stuck in the middle of it, and actually I don't know anything about them. Yeah, which wasn't ideal at the time.’ (P03)

‘So yeah, really user-friendly. The content was good. The only problems I suppose, but I guess it's just teething problems, [in relation to] how new the programme is, […] I remember they were saying when they were doing things like behavioural activation and using the planner, you couldn't set up recurring events for example in the diary or at least the person that I was working with couldn't. So they were finding it quite frustrating they were having to save they'd walked the dog at the same time every day when they were trying to plan it in, they were finding it frustrating having to put it in over and over again and they said they wished they could have done a bit of a, just a recurring entry instead. So just more things like that with the tools, they found some of them sent little reminders at the wrong time or yeah, little teething issues like that.’ (P012)

‘I think it always helps to have a smooth process, absolutely [.…] I think if there were kind of glitches and things initially I would imagine, I don't know but I would imagine that it would be quite difficult to keep some people kind of motivated and on track with that if they were, […] I imagine maybe something like that for some people might be quite frustrating or maybe something to stop them from continuing or wanting to continue with it.’ (P01)

### Flexibility to tailor b-CBT

Most PWPs reported a range of experiences in relation to their ability to tailor b-CBT to the patient's needs, in which they described various aspects of the task and goal activities as tailorable or inflexible.

#### F3 Tailoring of b-CBT

Some PWPs highlighted that their role within b-CBT enabled them to tailor the task, by overseeing, setting, framing and tailoring the patient's therapy across face-to-face sessions and i-CBT to better address the patient's goals. PWPs also suggested that they were at times able to adapt elements of activities pertaining to the task and bond in i-CBT (e.g. advising on the selection of modules). Face-to-face sessions in the clinic enabled the PWP to provide support and address emerging needs that could not necessarily be covered through the digital programme, enabling the PWP to offer the patient a wider selection of tasks to effectively help the patient achieve their goals:

‘I didn't go through all the modules with some people. So say for example the physical exercise one [optional module], if we didn't feel that was relevant we just spent another session really going over something like behavioural activation a little bit more. So for some patients we spent a lot more time focusing on a particular module and really making sure that was being understood, rather than just going through every module for the sake of it.’ (P012)

#### B3 Inflexible digital programme

PWPs conveyed that the digital component allowed limited scope for tailoring the task and addressing patients’ goals. For example, some PWPs noted that to unlock the final module, ‘relapse prevention’, the patient was required to complete the other three mandatory modules, which may not all have been relevant to them. This meant that patients who might have experienced rapid symptom resolution were still required to complete four core modules. PWPs also said that there was little opportunity to work transdiagnostically, since the content covered in the digital programme only addressed symptoms of depression, providing fewer opportunities to draw on tasks that addressed underlying causes that emerged during treatment. Although some PWPs addressed patients’ unmet needs in relation to their treatment goals in the clinic, others did not want to stray too far from the treatment protocol owing to concerns that tasks covered in face-to-face appointments could not be integrated with the task from the digital programme. Collectively this had a negative impact on the working alliance, as the PWP was unable to apply the most appropriate task to address the patient's goals:

‘I'd want to like not make it so […] strict so that, like I was talking about earlier with relapse prevention, like not having to complete all the modules to do that.’ (P04)

‘I think there was an element that was a little bit restrictive, I think because obviously sometimes if there's a mixed depression/anxiety and let's say, anxiety's forming a barrier, then things like relaxation exercises you obviously can't do that because it's not in the platform. I guess also knowing how MoodBuster goes through it, it goes through it from a very kind of classically “just depressed” state … ’ (P03).

‘[With] MoodBuster it comes back to that idea that we're kind of stuck sometimes with the things that are on there, so we'll go off-script, if you like, then I'm not actually utilising the programme. So there's been times when I've done relaxation or whatever in a session, which is part of a typical protocol for depression, but if it's come up, then we'd do it if it ever seems clinically relevant, and there's no way to incorporate that with MoodBuster for that session, because they didn't use it and I didn't give them any homework around it. And then again, that means that you can't then finish one of the modules in the time that we have, so it gets like a knock-on impact.’ (P04)

### Confidence in delivering b-CBT

Finally, PWPs also reported that their level of confidence in delivering b-CBT affected their ability to effectively build and maintain all components of a working alliance.

#### F4 Training and support

Some PWPs said that receiving training, having access to training resources and receiving technological support (e.g. related to patient log-in or technical issues) on how to use the i-CBT programme helped them feel more confident in delivering the task using the digital programme:

‘I think the training was really good and I was able to kind of spend time looking at the programme and looking at what was involved. [….] So, I think it was, I was well prepared for the session.’ (P15)

‘Well, a couple of times just with questions about things, more about things that had come up in sessions or questions I'd been asked [.…] so whenever I've needed to contact them [the research team] or ask anything I've always got a really quick response, really supportive.’ (P14)

‘I had quite a gap between having the training and then having a patient on the programme so I think the training was definitely helpful but I had to do a bit of a refresher beforehand.’ (P04)

#### B4 Low confidence and practice

Most PWPs reported feeling apprehensive owing to lack of confidence in delivering b-CBT and were unclear about their precise role within the blended format of delivery. PWPs said that their lack of expertise and experience in delivering the intervention made them feel anxious and hindered their confidence when introducing and delivering the task. A few PWPs also mentioned that they felt less control over their management of the patient's treatment because they were unclear about how well their patients understood or benefitted from the tasks on the i-CBT programme, limiting the PWPs’ ability to apply their judgement with respect to the selection of task to address a patient's needs and overarching goals and impeding their ability to effectively build a bond:

‘I found it a bit more difficult, because my confidence was a lot lower using this approach. So I was probably more in my head like, what am I supposed to be doing in this session, rather than actually being able to develop an alliance with the person in front of me. I don't think I had much of a therapeutic alliance with the specific person. Consequently, their engagement was really, really low.’ (P07)

‘Yeah, so … I think I still felt connected with them, […] I guess I felt like I had a little bit less control over exactly what they were doing because they were doing it on the modules … Possibly a good thing but then you also think ultimately, I guess you have that really kind of slightly arrogant view that you have to be the one to do this [deliver CBT], that only I can do this properly!’ (P03)

## Discussion

### General findings

Participants reported four facilitating factors in building a working alliance in b-CBT: expansion of time in treatment, having access to a wider toolkit, being able to tailor b-CBT, and receiving an appropriate level of training and support. Participants also reported four barriers to developing a working alliance: perceiving b-CBT as time and resource intensive, experiencing usability problems, not having the flexibility to tailor fixed elements of the digital programme to patients’ needs, and feeling a lack of confidence in delivering the b-CBT intervention.

The higher-order categories outlined in Fig. 2 highlight a spectrum of PWPs’ experiences of facilitators and barriers in building a working alliance. Facilitators such as ‘expansion of time’ and ‘access to a wider toolkit’ appear to enhance the PWPs’ ability to engage the patient with treatment activities beyond what would have been possible in only face-to-face therapy. Conversely, ‘flexibility to tailor the intervention’ and ‘training and support’ appear to lay the foundations that enable the working alliance to be effectively developed. Barriers such as ‘low confidence and practice’ and ‘time intensive’ were perceived as short-term problems that could be resolved over time as PWPs became adept in delivering the intervention. On the other hand, not being able to effectively tailor fixed programme features such as content and tools appeared to pose a long-term threat to the working alliance. ‘Programme usability problems’ may present both short-term and long-term threats to the working alliance, depending on whether usability or technical issues can be resolved.

### Evaluation in relation to other studies

Bordin's^10,11^ task and goals appear to be the most affected by therapists’ perceptions of the working alliance in a b-CBT context. This is expected considering that the therapeutic activities pertaining to the goals and task are predominantly accessed through the digital programme. Inflexible digital programme features and technical problems that affect patients’ engagement with the agreed goals and task appear to diminish PWPs’ role in collaboratively working on the agreed goals and task, and their role as a ‘major source of selection’ of the task.^10,11^ On the other hand, digital programme features were perceived to extend treatment beyond the clinic and offer a wider selection of tasks to address patients’ goals. Having in-person sessions appeared to be essential in reviewing and addressing unmet needs with respect to goals and task.^10,11^ Bordin's^10,11^ bond was also perceived to be affected by the b-CBT context, in which the i-CBT programme appeared to expand the time available for the PWP to work on the bond in clinic-based sessions. However, low confidence in delivering the intervention across PWP–patient shared activities appeared to undermine their capacity to forge an effective bond. Our findings also align with our previously described^18^ framework of patients’ working alliance demands in which a new component called usability heuristics outlines how functionality features (e.g. ‘access and immediacy of the task’ and ‘ease of use’) of the digital programme, and the capacity to offer personalised and complementary activities across in-person sessions and i-CBT, were critical in meeting patients’ goal, task and bond needs, as well as in patients’ ability to engage in supervised self-directed treatment.^18^

Our findings are supported by several qualitative studies that broadly explored therapists’ experience of delivering internet-based psychotherapies. Our findings in relation to the impact of ‘experiences of time’ and ‘flexibility in tailoring b-CBT’ on the working alliance were consistent with findings from a systematic review of health professionals’ perspectives on implementing internet-based therapies. The review found that guided and blended internet-based interventions were perceived to extend the time needed to develop a patient–therapist alliance, facilitate the building of rapport and allow the active monitoring and follow-up of patients.^27^ The importance of the digital programme's functionality and customisability was also highlighted in a qualitative study of therapists’ perspectives on barriers and facilitators in implementing b-CBT for depression conduced by a team at the German site of the E-COMPARED study.^19^ Findings revealed that persistent technical problems that could not be resolved caused ‘anger, frustration, and demotivation in both patients and therapists’, while ‘limited customisability and autonomy of decisions concerning blended therapy’ had a negative impact on the patient–therapist alliance.^19^

PWP concerns regarding case-load management that stemmed from additional commitments attributed to b-CBT were also consistent with other health professionals’ perspectives of implementing guided internet-based therapy, in which they emphasised the need for targeted training and organisational support to manage changed workflows and help therapists incorporate online therapies into their practice.^27^

The findings of our study appear to be relevant to broader implementation domains within the consolidated framework for implementation research (CFIR),^28^ such as: adaptability of the core components of the digital programme; and the implementation climate, which affects the time available for treating each patient; and the level of compatibility between the intervention and the workflow of the service. These implementation domains could therefore be specifically considered in relation to strengthening the working alliance in b-CBT.^28^

The barriers outlined in our study suggest that key competencies relating to the building of a working alliance during the delivery of CBT for people with depression and anxiety in IAPT services may be compromised. Table 2 highlights how the barriers outlined may have a negative impact on competencies relating to PWPs’ ability to: structure sessions and maintain appropriate pacing; manage obstacles to CBT therapy; use clinical judgement when implementing treatment models; adapt interventions in response to patient feedback; select and apply the most appropriate CBT method; and engage patients to foster and maintain a good therapeutic alliance.^29^ Future research is required to directly evaluate these factors.

**Table 2:** Working alliance-related competencies for delivering cognitive–behavioural therapy (CBT) for depression and anxiety that may be negatively affected by the working alliance barriers identified in the current study

Perceived working alliance barriers	Therapist competencies^29^ that may be at risk
Time intensive	Capacity to structure sessions and maintain appropriate pacing
Programme usability problems	Capacity to manage obstacles to cognitive–behavioural therapy (CBT)
Inflexible digital programme	Capacity to use clinical judgement when implementing treatment models Capacity to adapt interventions in response to patient feedback Sharing responsibility for session structure and content
Low confidence and practice	Ability to structure sessions Sharing responsibility for session structure and content Capacity to select and apply most appropriate behavioural therapy or CBT method Ability to engage a patient Ability to foster and maintain a good therapeutic alliance

### Strengths, limitations and future research

Although other studies have touched on the working alliance when exploring therapists’ general experiences of implementing i-CBT, our study is the first to conduct a focused investigation of PWPs’ experiences of the working alliance in a b-CBT intervention for depression, that delineates how the implementation of b-CBT can be used to strengthen the working alliance. A key methodological strength of our study involved adopting a guiding framework to understand the working alliance in b-CBT. Bordin's^10,11^ conceptualisation of the working alliance and a more recent framework of the working alliance in b-CBT for depression^18^ was used to ensure that barriers and facilitators were theoretically driven. Adopting two qualitative interview approaches (individual interviews and FGDs) enabled both in-depth analysis and opportunities to confirm shared insights in a group setting.^23^

Several limitations should be noted. Our sample (*n* = 13) was small owing to the limited number of PWPs meeting the inclusion criteria, the high number of PWPs leaving services before they were due to be interviewed (*n* = 7) and time constraints concerning data collection during the trial. The PWPs saw three patients on average, indicating little experience in delivering b-CBT prior to the interviews. Moreover, 40% of the PWPs had a bachelor's degree level of experience, and one PWP only had 12 months of experience, highlighting that some PWPs were at the low end of the experience spectrum. It is therefore possible that barriers pertaining to low confidence may have been resolved over time and with practice. Nevertheless, understanding short-term barriers could ensure that PWPs are effectively supported to use the full breadth of the digital programme's features. Two PWPs who took part in individual interviews were also later involved in FGDs, which may have affected the data generated in the FGDs. The emergence of COVID-19 might have resulted in PWPs becoming more familiar with and confident in using online therapeutic platforms, compared with when data collection took place (2015–2017). Only one type of digital programme and blended sequence was used in the study, which may have limited responses elicited on the working alliance. Taken together, these limitations may reduce the generalisability of our findings.

Despite these limitations, our findings appear to be sufficiently supported by studies using different digital programmes, delivery formats and mental healthcare professionals, thus lending greater confidence to our findings.^18,19,27,30,31^ Future research should build on our study, to develop an implementation checklist that can be used to support services in optimising PWPs’ experience in forming a working alliance.

### Implications

The importance of the working alliance in psychotherapy appears to also extend to treatments that incorporate digital technologies.^32^ ‘Common elements of therapy’, which encompass the working alliance, has been identified as a key research priority for digital technologies in mental healthcare.^9^ With an increase in the use of digital technologies in mental healthcare, our findings may help PWPs and services navigate a hybrid format of delivery, to effectively harness and preserve a central mechanism of change in psychotherapy.

Our findings suggest that the working alliance in b-CBT can be enhanced in three ways. First, interventions and service workflows should align in terms of duration and frequency of sessions. Additional PWP duties in b-CBT (e.g. becoming familiar with the content of the programme, therapist portal activities such as sending messages to the patient or reviewing progress) should be considered when estimating the duration of the intervention, and the PWPs’ overall case-loads should be taken into account to ensure that they are able to deliver the task effectively and leverage the full breadth of tools available on the programme to form a good working alliance. Second, the digital programme should offer transdiagnostic tools and enable flexible and adaptable features (e.g. being able to choose the sequence and number of modules the patient completes) to enable the PWP to effectively tailor the intervention to their patient's needs. Third, PWPs should be provided with appropriate support in relation to the digital programme. As the use of digital tools in mental healthcare increases, so does the burden on the PWP, who will be required to learn how to operate multiple digital tools, manage patients’ activities online and resolve technical problems. Wisniewski & Torous^33^ have proposed a new role within care teams called ‘digital navigators’ to reduce the time pressure on PWPs. This is particularly important considering that numerous studies have reported high rates of stress and burnout among PWPs,^34,35^ which can lead to PWPs’ avoidance of the patient–therapist alliance.^36^ The digital navigator's role would involve setting up the programme, troubleshooting technological problems, reviewing patient data and producing data summaries, to provide PWPs with additional sights and time to effectively form a working alliance.^33^

## Data Availability

The data that support the findings of this study are available on reasonable request from the corresponding author, A.D.

## Supplementary material

For supplementary material accompanying this paper visit http://doi.org/10.1192/bjo.2022.546. click here to view supplementary material
