# Supplementary material for: Practitioners’ experience of the working alliance in a blended cognitive–behavioural therapy intervention for depression: qualitative study of barriers and facilitators
Source: BJPsych Open. 2022 Jul 25;8(4):e142. doi: 10.1192/bjo.2022.546 (PMC9344874; doi:10.1192/bjo.2022.546)
Supplement: Supplementary file 1 [file S2056472422005464sup001.docx]

**Supplementary Material**

Contents

[Topic guide 1: Individual Interviews 1](#_Toc96722403)

[Topic guide 2: Focus group discussions 4](#_Toc96722404)

# Topic guide 1: Individual Interviews

[Instructions: Special efforts should be taken to cover different perspectives using prompts in the topic guide or as necessary]

**Warm-up questions**

Examples:

1. Could you remind me of how many patients you have seen in the blended therapy arm?
2. Could you tell me about your experience of blended therapy with your most recent patient?

**Open Questions**

1. Can you tell me about your experience of delivering blended therapy?

- Was there anything that surprised you about it?
  - In a good way?
  - In a bad way?
- How did your patients respond?
- How did this compare to delivering face-to-face therapy?
- How did you feel about using the blended intervention?
- After you used it for the first time, did you feel you wanted to try it again?
- Did you feel it was useful for your patients?
- Did you feel supported and encouraged by your managers to use it?

1. Can you tell me about your initial expectation of delivering blended therapy?

- How did you think your patients were going to respond?
- Did you think this was a good idea or something that you wanted to try?
- Did you think it was too complex and a bit of a burden?
- Did you feel confident you would be able to use it?

1. At any point during your sessions, did you experience any difficulties, problems, or setbacks?

- Can you give me some examples? [make a list]
- Can you tell me what happened when ‘X situation took place’ … [review all situations]?
- Why was this difficult?
- Did it get resolved?
- Can you tell me how things were resolved? OR I would be interested to know why you *think x situation* wasn’t resolved.

1. Based on your experience, could you tell me what you think the advantages are of using a blended approach?
2. Based on your experience, could you tell me what you think the disadvantages are of using a blended approach?

**Specific Questions**

1. I would like to know about your experience of using the Moodbuster platform?

- What did you think about the platform?
- Was this helpful for your practice?
- Was this helpful for your patients?
- Is there anything that could have been included that would have been helpful to you or your patients?
- Was there anything challenging that you experience?
- Did you feel confident from the start that you knew enough about how to use the platform?

1. Did you experience any problems when using the platform?

- Could you tell me more about some of the problems you’ve experienced?
- How did this affect therapy?
- (if so) how did this make you feel ….?
- How did you cope/ or deal with these problems?
- Did this affect your attitude towards the platform?

1. Can you tell me about how the patients goals were established?

- What helped?
- Were the patient goals static throughout therapy or did they change?
- What hindered your ability to …do this?
- Was there any differences in the way goals were set between blended and face-to-face therapy?

1. Generally speaking, how well were you able to engage your patient to the modules of Moodbuster?

- How were the tasks selected?
- What did you think about this approach?
- How does this compare to engaging patients to face-to-face therapy?
- Did you notice any benefits?
- Did you check with patients if they are using Moodbuster?

1. Can you tell me about your experience of building an alliance or relationship, with your patient?

- What helped?
- What hindered your ability to… do this?
- How does this compare to patients in face-to-face therapy?
- Does the computerised component affect how you engaged with the patient?
- How so?

1. Would you say you felt committed to delivering blended therapy?
   - What helped?
   - What prevented this?

**Ending Questions**

---------------------------------------Provide summary--------------------------------------------

1. If you had to change one or more things about the intervention, what would it be?
2. Do you think there is room for this type of blended intervention in IAPT?
3. Outside of the trial, would you consider using a blended approach with prospective patients?
4. I have one last question, on a whole, how good do you think you are at using technology?

Thank and close

# Topic guide 2: Focus group discussions

[Instructions for interviewers: Special efforts should be taken to cover different perspectives using prompts in the topic guide or as necessary]

Focus group discussions topic guide

1. What was your experiences of blending e-interventions with therapy?

- What is your view on the blended therapy approach within psychological treatments?
- Can you tell me if you have or had any reservations about using this approach?

1. Can you tell us if or how you blended the treatment?

- Did you try different ways, what worked better?

1. Did you feel prepared and supported to carry out the treatment?

- Did you feel supported by the service and research team?

1. What do you think about Moodbuster?

- Was it user friendly?
- Did it have the right content?
- Did you encounter any problems with the platform?
- What do you see as the advantages of Moodbuster to you or your patients?

1. How does Moodbuster compared to other platforms that you’ve used?

- Do you think we’ve used Moodbuster to its full potential?
- Do you think it could have been implemented more effectively?
- if so, how?

1. Generally speaking, how well were you able to engage your patient to the modules of Moodbuster?

- How were the tasks selected?
- What did you think about this approach?
- How does this compare to engaging patients to face-to-face therapy?
- Did you notice any benefits to this approach?
- Did you check with patients if they are using Moodbuster?

1. How were the patients goals established?

- What helped?
- What hindered your ability to do this?
- Were the patient goals static throughout therapy or did they change?
- Was there any difference in the way goals were set between blended and face-to-face therapy?

1. Can you tell me about your experience of building a therapeutic (working / alliance) relationship, with your patient?

- What helped?
- What hindered your ability to… do this?
- How goes this compare to patients in face-to-face therapy?
- Does the computerised component affect how you engaged with the patient?
- How so?

1. Would you say you felt committed to delivering blended therapy?
   - Do you feel your attitudes to blended therapy have changed?
2. Is there anything you wanted to mention that we didn’t get a chance to talk about?

Thank and close
